# AC2F: A Lightweight Adaptive Pursuit Strategy for UAVs in Complex Public Domains with Real-World Validation

**DOI:** 10.3390/s26123790

**Published:** 2026-06-14

**Authors:** Hangtao Zhang, Fanglin Zhou, Yuntao Xue, Yunze Xue

**Affiliations:** 1 College of Electronic and Information Engineering, Taiyuan University of Technology, Taiyuan 030024, China; zhanghangtao@link.tyut.edu.cn (H.Z.); zhoufanglin@link.tyut.edu.cn (F.Z.); 2 School of Mechanical Engineering, Tsinghua University, Beijing 100084, China; xueyz23@mails.tsinghua.edu.cn

**Keywords:** multi-UAV pursuit, Apollonius circle, Bayesian optimization, adaptive dynamic window approach (DWA), lightweight computing

## Abstract

Executing multi-UAV cooperative pursuit in complex public domains requires balancing interception efficiency with flight safety under strict micro-platform constraints. Existing planners often struggle with high computational overhead or lack kinodynamic adaptability in heterogeneous environments. To address this, we propose AC2F, a lightweight Adaptive Coarse-to-Fine hybrid framework featuring a bidirectional state-switching mechanism. The framework utilizes the Apollonius circle for efficient global guidance during the coarse phase, dynamically transitioning to a Dynamic Window Approach (DWA) upon detecting path oscillations or entering terminal capture zones. To ensure robustness, a dual-layer parameter paradigm integrates offline Bayesian optimization for globally optimal baselines with online real-time weight adaptation based on target distance. Extensive simulations show that AC2F effectively escapes local minima, such as urban-style U-shaped traps. Real-world suburban validation confirms an 86% capture rate with minimal computational overhead, demonstrating AC2F’s suitability for public domain protection and civil security.

## 1. Introduction

Cooperative multi-UAV pursuit [1,2] has emerged as a cornerstone for low-altitude public safety maintenance, security surveillance [3], and civilian infrastructure protection. As uncrewed aerial systems become increasingly integrated into public domains, the ability to autonomously intercept non-compliant targets has become a critical requirement for modern urban management. However, deploying these cooperative systems on micro-UAVs—the preferred choice for flexible civilian deployment—highlights a critical bottleneck: computational constraints. While mainstream end-to-end deep reinforcement learning (DRL) [4] algorithms exhibit excellent theoretical performance, their practical engineering implementation in complex social environments remains highly challenging. DRL models demand significant onboard computing power [5,6], a requirement that is further exacerbated in multi-UAV scenarios where cooperative communication processing consumes substantial resources at the expense of flight endurance. Consequently, there is an urgent need to develop a path planning algorithm that delivers high efficiency with a minimal computational burden to ensure operational reliability and regulatory compliance in next-generation civil aviation applications.

Current obstacle avoidance strategies face an inherent dilemma between global optimality and local reactivity. Global planning algorithms, such as A* [7] and Rapidly-exploring Random Trees (RRT), are limited by low replanning frequencies in large-scale dynamic environments. Conversely, traditional local methods like Artificial Potential Fields (APF) are computationally efficient but highly susceptible to local minima (e.g., U-shaped traps) when deployed as low-level motion controllers. Furthermore, they often generate high-frequency control oscillations, failing to satisfy the kinodynamic requirements for high-speed, smooth UAV flight. While DRL models bypass some of these control issues, their computational footprint precludes micro-UAV deployment. The Dynamic Window Approach (DWA) therefore provides a viable kinodynamic alternative, but its performance depends strongly on the weight coefficients in its evaluation function [8]. Conventional heuristic tuning is labor-intensive and often sub-optimal [6], especially in multidimensional parameter spaces with heterogeneous obstacles.

To balance global interception efficiency and local flight safety, this paper proposes an Adaptive Coarse-to-Fine (AC2F) hybrid planning framework. This framework introduces a bidirectional state-switching mechanism that overcomes the limitations of traditional unidirectional pipelines. By default, the pursuit UAV system operates in the coarse guidance stage, utilizing the Apollonius circle to calculate and predict interception points, supplemented by lightweight reactive avoidance to maintain basic safety. The system continuously monitors the UAV’s state; upon approaching the target (triggered by a distance threshold) or detecting entrapment in a local minimum (e.g., prolonged in-place oscillation), it immediately transitions to the fine control stage. In this stage, the DWA [8] is employed to achieve smooth obstacle avoidance while satisfying kinodynamic constraints [9]. To address the issues of tracking failure or instability caused by fixed DWA parameters, we further design a dynamic parameter adjustment mechanism. Moreover, to prevent the system from being trapped in local optima and to maintain responsiveness to the global situation, the fine control stage operates within a short, sustained time window, after which it automatically reverts to the coarse stage. This creates a dynamic closed loop between global planning and local obstacle avoidance. For the fine control stage, the optimal baseline parameters are obtained via offline Bayesian optimization [10,11].

The contribution of AC2F is therefore positioned as a task-specific hybrid pursuit architecture rather than a new standalone version of Apollonius guidance, APF, DWA, or Bayesian optimization. Its novelty lies in coupling these components through a bidirectional finite-state interface designed for cooperative pursuit: analytical interception geometry supplies a low-cost global intent, oscillation-aware DWA is invoked only when the local topology requires kinodynamic reasoning, and the controller returns to the coarse layer after a bounded local planning window. This design targets a deployment gap not fully addressed by general hybrid path planners, namely real-time multi-UAV pursuit on micro-platforms where target motion, obstacle avoidance, and onboard computation must be balanced simultaneously.

The main contributions of this paper are summarized as follows:

- A lightweight AC2F hybrid planning architecture for public domains: We propose a bidirectional Adaptive Coarse-to-Fine (AC2F) framework that integrates analytical geometric guidance with kinodynamic planning, specifically designed to handle the tight computational constraints of micro-UAVs in complex environments.
- A robust switching mechanism with oscillation detection: We introduce a bidirectional state transition logic based on oscillation detection and time-window callbacks, enabling the system to effectively escape local minima such as urban-style U-shaped traps.
- A dual-layer parameter optimization paradigm: We present a systematic approach combining offline Bayesian optimization for global baseline parameters with online real-time weight adaptation to ensure mission-critical reliability under highly dynamic pursuit–evasion states.
- Real-world validation in suburban scenarios: The practical engineering feasibility of the framework is demonstrated through flight tests, confirming its minimal computational footprint and suitability for next-generation civil security tasks.
- Reproducible implementation and statistical validation: We summarize the switching thresholds, DWA sampling parameters, memory penalty, BO configuration, numerical-simulation protocol, and onboard latency measurements to make the reported performance traceable and reproducible.

## 2. Related Work

### 2.1. Geometric Guidance and Cooperative Pursuit

Classical pursuit–evasion strategies, such as Proportional Navigation Guidance (PNG) and Pure Pursuit, have been extensively studied for their ability to generate rapid guidance commands directly via line-of-sight (LOS) [12] feedback. In cooperative multi-UAV scenarios, the Apollonius circle method is particularly prominent [13,14]. By leveraging the velocity ratio between pursuers and evaders, it analytically constructs an inevitable interception zone, allowing for the instantaneous calculation of optimal capture points.

Despite their theoretical optimality and exceptional computational efficiency in open airspace, these geometric methods are fundamentally open-loop strategies based on point-mass assumptions [2,15]. They inherently lack environmental awareness. Consequently, relying exclusively on geometric guidance in obstacle-dense environments introduces severe collision risks, necessitating the integration of reactive navigation modules.

### 2.2. Reactive Obstacle Avoidance for UAVs

To mitigate the safety deficiencies of pure geometric tracking, local reactive planners are conventionally employed. Artificial Potential Fields (APF) remain a popular choice due to their simple mathematical formulation and minimal computational footprint. However, APF is highly susceptible to local minima [16]. When navigating complex topological structures, such as U-shaped traps or narrow corridors, APF frequently induces high-frequency control oscillations, rendering the UAV aerodynamically unstable.

As a more physically grounded alternative, the Dynamic Window Approach (DWA) [8,17] samples feasible trajectories directly within the velocity space (v,ω). By strictly enforcing the robot’s kinodynamic constraints—such as maximum velocity and acceleration limits—DWA generates inherently smooth and executable trajectories. While DWA is a standard in ground-based automated guided vehicles (AGVs), its deployment in agile, three-dimensional UAV pursuit remains sparse. Existing UAV navigation frameworks tend to favor optimization-based approaches like Model Predictive Control (MPC) for kinodynamic planning, frequently overlooking the low-latency and lightweight deployment advantages that DWA inherently offers.

Recent UAV navigation studies have begun to adapt DWA to aerial swarms, for example by combining 3D vector field histogram sensing with dynamic-window velocity selection [17]. In parallel, MPC-style and optimization-based planners have demonstrated strong trajectory quality for quadrotor swarms in cluttered spaces [18], but they typically require denser local optimization and more elaborate state prediction than the controller targeted in this work. Lightweight learning-based navigation methods, including self-supervised systems and DRL-based UAV navigation, provide an alternative route to adaptation [5,6]; however, their training and verification requirements remain non-trivial for safety-critical pursuit. AC2F is positioned between these lines: it keeps DWA’s explicit kinodynamic feasibility while using geometric pursuit information and offline parameter search to avoid a heavy online optimizer.

### 2.3. Parameter Adaptability in Kinodynamic Planning

A critical bottleneck in applying DWA to highly dynamic pursuit missions lies in its evaluation function. Conventional DWA implementations utilize fixed heuristic weights to score heading alignment, obstacle clearance, and velocity. In high-speed pursuit, however, the UAV’s navigational priorities are highly transient: long-distance tracking requires a strict heading lock (heading-prioritized), terminal capture demands maximum acceleration (velocity-prioritized), and navigating through dense clutter mandates absolute safety margins (clearance-prioritized).

Fixed-weight configurations fail to reconcile the fundamental conflict between “aggressive capture” and “conservative avoidance.” This rigidity often results in suboptimal behaviors, such as premature deceleration during target approach or collisions due to insufficient reactive margins. Furthermore, tuning these weights traditionally relies on exhaustive trial-and-error, a process that is not only labor-intensive but also struggles to identify global optima across multidimensional parameter spaces. The absence of a systematic, computationally efficient parameter adaptation mechanism remains a significant hurdle.

### 2.4. Hybrid Path Planning Architectures

To synthesize global optimality with local safety, hybrid planning architectures have become the prevailing standard. Common paradigms, such as A* or Rapidly-exploring Random Trees (RRT) [19] paired with APF, utilize graph-search algorithms for global pathfinding and reactive methods for local smoothing.

Nevertheless, continuous replanning with traditional global algorithms imposes an unsustainable computational burden on micro-UAVs, particularly when tracking evasive moving targets. To bridge this gap, we propose a lightweight hybrid architecture integrating geometric guidance with an adaptive DWA. By substituting time-intensive graph searches with the analytical Apollonius circle, and replacing the oscillation-prone APF with a kinodynamically robust, dynamically weighted DWA, our framework preserves the low-latency nature of geometric methods while rigorously ensuring flight safety and capture efficiency [17,18].

Compared with general hybrid planners whose global layer solves a static or quasi-static path-planning problem, AC2F treats the global layer as a pursuit–evasion guidance problem and uses the Apollonius capture set as the task-level reference. Compared with single-direction coarse-to-fine pipelines, its backward transition from DWA to geometric guidance prevents prolonged local myopia after the UAV escapes a trap. This bidirectional scheduling rule is the main architectural distinction evaluated in the ablation experiments.

## 3. Problem Formulation

To establish a computationally efficient and rigorously defined foundation for the multi-UAV pursuit task, this study formulates the environment and the associated kinematics within a two-dimensional (2D) planar space.

To guarantee that the generated trajectories strictly adhere to the physical capabilities of the UAVs, we model the system based on unicycle kinematics, explicitly incorporating non-holonomic constraints. Let the state vector of the UAV in the global coordinate system be defined as $x={\left[x,y,\theta \right]}^{T}$, where *x* and *y* represent the planar position and θ denotes the heading angle. The control input vector is defined as $u={\left[v,\omega \right]}^{T}$. The continuous-time kinematic model of the UAV is expressed as follows:

(1) $$\left[\begin{array}{c} \dot{x}\\ \dot{y}\\ \dot{\theta } \end{array}\right]=\left[\begin{array}{cc} \cos\theta & 0\\ \sin\theta & 0\\ 0 & 1 \end{array}\right]\left[\begin{array}{c} v\\ \omega \end{array}\right]$$

Furthermore, the physical actuation limits of the micro-UAV platform impose the following hard constraints on the system:

(2) $$\left\{\begin{array}{c} 0\leq v\leq v_{max}\\ \left|\omega \right|\leq \omega_{max}\\ |\dot{v}|\leq a_{max},\;\left|\dot{\omega }\right|\leq \alpha_{max} \end{array}\right.$$

where *v* and ω denote the linear and angular velocities, respectively. The parameters $v_{max}$ and $\omega_{max}$ are the upper bounds of these velocities, while $a_{max}$ and $\alpha_{max}$ represent the maximum allowable linear and angular accelerations. These intrinsic kinodynamic constraints will be explicitly integrated into the velocity sampling space of the subsequent DWA module to ensure absolute trajectory feasibility.

## 4. Methodology

### 4.1. Overall Framework

**Figure 1:**
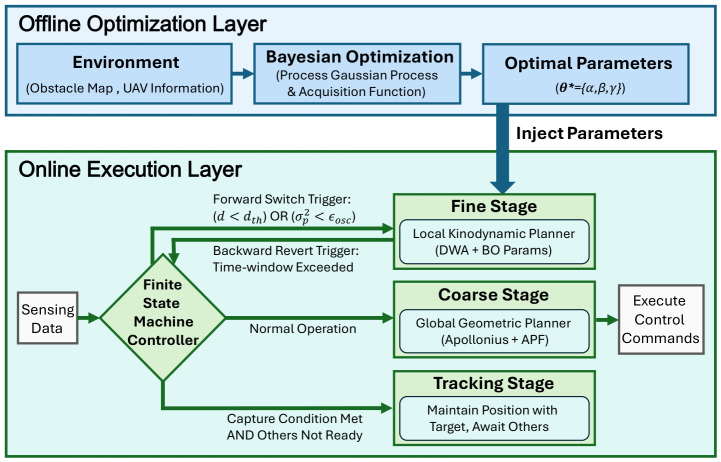
Overall framework of the proposed AC2F strategy.

AC2F is organized into two coupled layers: offline parameter optimization and online execution, as shown in Figure 1. This structure separates computationally intensive tuning from the flight loop while retaining onboard adaptability for resource-constrained multi-UAV pursuit.

- Offline Optimization Layer:Bayesian Optimization (BO) is used before deployment to identify the baseline DWA weights. Environmental features and UAV kinematic limits are evaluated through a Gaussian Process (GP) surrogate and an acquisition function, yielding $\theta^{*}=\left\{\alpha ,\beta ,\gamma \right\}$ for onboard initialization.
- Online Execution Layer: The onboard layer is governed by a Finite State Machine (FSM). Using target distance, oscillation status, and capture synchronization, the FSM selects one of three states before sending commands to the low-level controller:
  – Coarse Stage (Global Geometric Planner): This default state uses Apollonius circle geometry to generate the global pursuit reference and lightweight APF avoidance to handle sparse obstacles with low computational cost.
  – Fine Stage (Local Kinodynamic Planner): The FSM enters this state when the UAV approaches the target ($d<d_{th}$) or shows local oscillation ($\sigma_{p}^{2}<\epsilon_{osc}$). The DWA planner then performs kinodynamic obstacle avoidance using the BO-optimized weights. If the fine-stage time window expires before capture, the controller returns to the coarse stage to restore the global pursuit reference.
  – Tracking Stage: When one UAV satisfies the capture condition before its partners are ready, it holds its relative position to the evader until synchronized encirclement can be completed.

Thus, the framework limits online computation to state switching, geometric guidance, and local trajectory scoring, which supports real-time execution on micro-UAV processors.

Table 1 summarizes the implementation parameters used in both numerical simulations and field tests. The distance and oscillation thresholds were selected to activate the fine planner before terminal capture and to detect repeated local motion without responding to normal curved pursuit. Unless otherwise stated in the ablation study, the same parameter set was used for all repeated trials.

The state transitions and their activation conditions are summarized in Table 2.

### 4.2. Coarse Stage: Ideal Geometric Guidance

The coarse stage reduces online planning cost by separating cooperative guidance from local avoidance. Based on the kinematic differences between the pursuers and the evader, the Nash equilibrium formulation computes the worst-case global interception point $P_{global}$, which serves as the predictive encirclement destination. Lightweight APF control is then used for reactive avoidance during the approach.

#### 4.2.1. Apollonius Circle-Based Geometric Collaborative Guidance

The Apollonius circle, a geometric model describing the locus of points with a given distance ratio from two fixed points, effectively represents the reachable sets and dominant capture zones of both parties in a pursuit–evasion task. By solving for the Nash Equilibrium of the union of multiple circles, we establish a globally advantageous target point for the subsequent fine control stage.

In a 2D workspace, let the position of the escaping target be $p_{e}\in R^{2}$ and the position of the *i*-th pursuing UAV be $p_{i}\in R^{2}$. The velocity ratio between the pursuer and the evader is defined as $\lambda_{i}=\frac{v_{i}}{v_{e}}$ (where $\lambda_{i}>1$ indicates a speed advantage for the pursuer). The set of points p satisfying this specific distance ratio forms the Apollonius circle, and its defined capture region $A_{i}$ is expressed as:

(3) $$A_{i}=\left\{p\in R^{2}\;|\;∥p-p_{i}∥\leq \lambda_{i}\;∥p-p_{e}∥\right\}$$

Given the limited absolute control zone (the interior of the Apollonius circle) of a single UAV is limited, the capture region dynamically shrinks as the pursuit progresses. To satisfy the cooperative constraint of “synchronized multi-UAV suppression,” the geometric union of multiple capture regions must be utilized to form a closed encirclement. Taking a dual-UAV system as an example, let the Apollonius circles of Pursuer 1 and Pursuer 2 be $A_{1}$ and $A_{2}$, respectively. The effective collaborative capture region set $\Omega_{c}$, which simultaneously suppresses the evader under their joint action, is defined as:

(4) $$\Omega_{c}=A_{1}\cup A_{2}=\left\{P|P\in A_{1}\;\mathrm{or}\;P\in A_{2}\right\}$$

**Figure 2:**
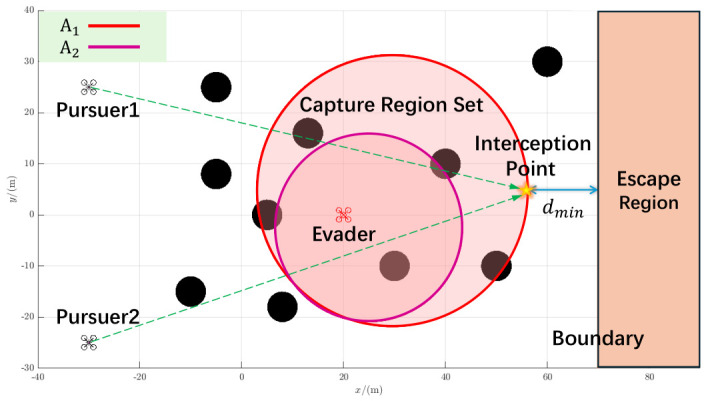
Diagram of the Apollonius Circle-Based Geometric Collaborative Guidance.

As illustrated in Figure 2, the core conflict of the pursuit–evasion game is as follows: the evader attempts to cross the boundary *L* of the safe escape region, while the pursuer swarm strives to block this escape route. Let d(P,L) be the orthogonal distance from a spatial point *P* to the boundary *L*. Within the collaborative capture set $\Omega_{c}$, a typical zero-sum game emerges:

- **Evader’s Strategy:** Seek the breakthrough point closest to the safe boundary, i.e., $\min_{P\in \Omega_{c}}\;d\left(P,L\right)$.
- **Pursuers’ Strategy:** Force the evader to be as far as possible from the boundary before being captured, i.e., $\max_{P\in \Omega_{c}}\;d\left(P,L\right)$.

The Nash Equilibrium of this game represents the optimal predictive interception point $P_{global}$ for dual-UAV collaboration. This point is the tangent on the boundary of the joint capture set $\Omega_{c}$ that yields the minimum distance $d_{min}$ to the safe boundary:

(5) $$P_{global}=\arg\min_{P\in \Omega_{c}}\;d\left(P,L\right)$$

At this equilibrium point, the evader reaches its maximum escape distance under its current kinematic constraints, while the pursuers achieve the optimal timing for intervention. Faster UAVs can proactively maneuver to this point to blockade the area until the cooperating units arrive, thereby finalizing a hard encirclement.

#### 4.2.2. Coarse-Stage Reactive Collaborative Obstacle Avoidance

During the convergence of multiple UAVs toward the equilibrium point $P_{global}$, traditional independent Artificial Potential Fields (APF) can easily cause the swarm to break its cooperative formation when avoiding local obstacles, potentially leading to mission failure. Therefore, we propose a “collaborative-posture-driven” coordinated obstacle avoidance mechanism.

This mechanism applies virtual force fields to both obstacles and the target simultaneously, guiding the swarm to synchronously press toward the optimal interception point $P_{global}$. As a UAV approaches an obstacle, the non-linear repulsive potential field reshapes its local heading, producing a fluid-like, macroscopically collaborative avoidance posture. The repulsive force field $F_{\mathrm{obstacle}}\left(p\right)$ exerted by the *j*-th obstacle on a spatial point p is defined as:

(6) $$F_{\mathrm{obstacle}}\left(p\right)=\sum_{j}\eta \;\frac{p-p_{\mathrm{obs},j}}{∥p-p_{\mathrm{obs},j}∥}\cdot \frac{1}{{∥p-p_{\mathrm{obs},j}-r_{\mathrm{obs},j}∥}^{2}}$$

where $p_{\mathrm{obs},j}$ is the center-of-mass coordinate of the obstacle, $r_{\mathrm{obs},j}$ is the expansion radius of the obstacle (incorporating the UAV’s safety margin), and η is the repulsive gain coefficient. This modified potential field exhibits a strict asymptotic safety property near the obstacle boundary:

(7) $$\lim_{∥p-p_{\mathrm{obs},j}-r_{\mathrm{obs},j}∥\to 0}∥F_{\mathrm{obstacle}}\left(p\right)∥=\infty$$

**Figure 3:**
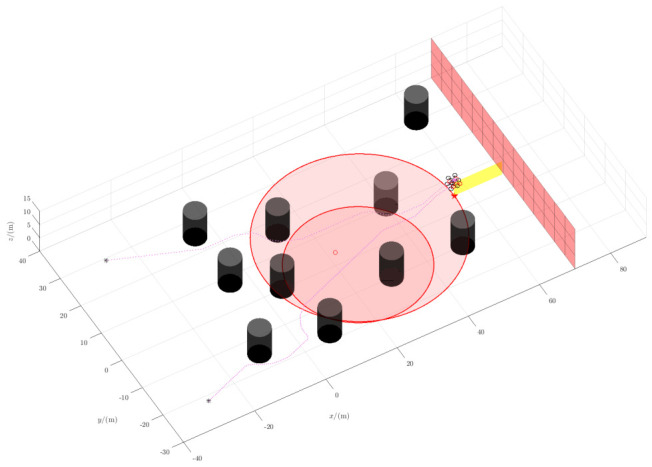
Pure APF baseline test demonstrating obstacle avoidance characteristics. The magenta dotted curves show the pursuer trajectories, the black cylinders denote obstacles, the red circles indicate the cooperative capture regions, the five-pointed star marks the predicted interception point, and the shaded box denotes the escape region.

This limit characteristic theoretically guarantees that the UAV trajectory will not penetrate the obstacle region, as demonstrated by the pure APF baseline test in Figure 3.

However, due to the inherent local minimum flaws of the APF, UAVs may experience path oscillation in complex canyons or dense obstacle zones. To ensure mission robustness, the system introduces a positional variance monitor. If the statistical variance of the UAV’s position within a sliding time window $t_{w}$ satisfies $\sigma_{p}^{2}<\epsilon_{osc}$ (where $\epsilon_{osc}$ is the oscillation tolerance threshold), and the capture condition has not yet been triggered, the control framework proactively intervenes. It smoothly transitions to the Fine Stage based on the FSM logic outlined in Section 4.1. In this stage, a kinodynamically constrained local optimizer is invoked to facilitate rapid escape.

### 4.3. Fine Stage: Kinodynamic Local Planning

The fine stage uses an improved DWA for local trajectory generation. Because DWA performance is sensitive to the evaluation weights (α,β,γ), AC2F combines offline BO-based baseline tuning with online distance-based adaptation, as detailed in Section 4.4.

Key enhancements to this stage include:

- **Introduction of a Memory Penalty:** A Memory penalty discourages candidate trajectories that return toward recently visited positions. This helps the UAV leave U-shaped traps or other local-minimum regions instead of repeatedly sampling similar motions.
- **Dual-Mode Adaptation of Time Horizon and Weights:** The planner switches between Approach Mode and Terminal Capture Mode according to $d_{target}$. Shortening $T_{pred}$ and increasing the velocity weight γ near the target balances stable long-range tracking with terminal capture accuracy.

#### 4.3.1. Trajectory Evaluation Function

**Figure 4:**
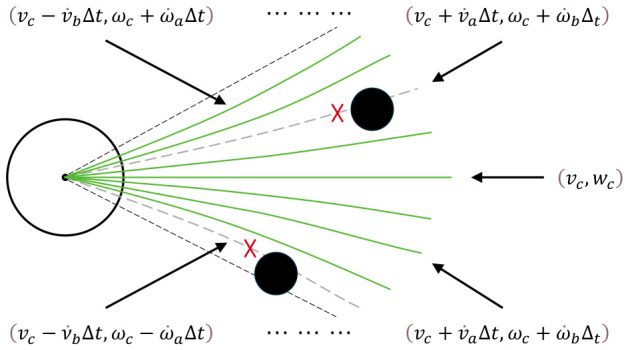
Trajectory evaluation function in DWA. Green curves denote collision-free candidate trajectories, gray dashed curves denote rejected trajectories, red crosses mark predicted collision points, black disks denote obstacles, and black dashed lines bound the sampled dynamic window.

For each sampled velocity pair (v,ω), the system predicts its trajectory over a future time window $T_{pred}$ and scores it using the evaluation function G(v,ω), as illustrated in Figure 4. The velocities and accelerations corresponding to the highest-scoring trajectory are then executed.

(8) G(v,ω)=α·Heading+β·Clearance+γ·Velocity−δ·Memory

The functions and roles of each sub-component are detailed as follows:

1. **Heading Function:**
  (9) $$\mathrm{Heading}\left(v,\omega \right)=\pi -|\Theta_{traj}-\Theta_{target}|$$
  Role: Measures the deviation between the orientation at the end of the predicted trajectory and the line-of-sight angle to the evader. A smaller deviation yields a higher score. This guides the UAV to correct heading errors caused by obstacle avoidance during the coarse stage, ensuring the nose consistently points toward the target.
2. **Clearance Function:**
  (10) $$\mathrm{Clearance}\left(v,\omega \right)=\min\left({\mathrm{dist}}_{min},D_{sat}\right)$$
  Role: Evaluates the Euclidean distance ${\mathrm{dist}}_{min}$ between the trajectory and the nearest obstacle.
  - Hard Constraint: If ${\mathrm{dist}}_{min}\leq R_{collision}$, the trajectory is flagged as a collision, scoring −∞, and is subsequently discarded.
  - Saturation Constraint: A safety distance saturation threshold $D_{sat}=2.0\;m$ is introduced. When the distance to an obstacle exceeds this threshold, the clearance score ceases to increase. This prevents the UAV from losing track of the target by abruptly diverting into wide-open spaces in pursuit of excessive safety margins.
3. **Velocity Function:**
  (11) Velocity(v,ω)=v
  Role: Directly rewards high-speed flight. Provided that obstacle avoidance and heading constraints are met, this compels the UAV to approach the target at a speed as close to $v_{max}$ as possible, thereby minimizing capture time.
4. **Memory Penalty:**
  (12) $$\mathrm{Memory}\left(v,\omega \right)=\sum_{i=1}^{N}f\left(\mathrm{dist}\left(P_{i},\mathrm{history}\right)\right)$$
  Role: Calculates the proximity of the predicted trajectory points to the UAV’s historical path. If the trajectory loops back toward visited areas, the penalty δ is amplified, compelling the UAV to break out of local optima traps, such as U-shaped obstacles.

#### 4.3.2. Adaptive Strategy and Weight Selection

Unlike traditional fixed-weight DWA, this paper proposes an adaptive parameter adjustment strategy based on target distance, designed to dynamically balance tracking stability and terminal capture capability. Depending on the distance $d_{target}$ between the UAV and the escaping target, the system transitions smoothly between two modes:

1. **Approach Mode**
  When the UAV is far from the target, the planner uses a longer prediction horizon $T_{pred}$, a larger heading weight α, and a smaller velocity weight γ. This setting prioritizes stable target bearing recovery after obstacle avoidance and supports continuous long-range tracking.
2. **Terminal Capture Mode**
  When the UAV enters the close-range capture zone, the planner uses a shorter prediction horizon and swaps the heading and velocity weights. This setting reduces overshoot-induced heading oscillation and helps reduce terminal distance error.

The online update rule is implemented as a deterministic lookup from the target distance rather than as an additional optimizer. Let $\theta^{BO}=\left(\alpha_{0},\beta_{0},\gamma_{0}\right)$ be the BO-derived baseline. When $d_{target}\geq d_{th}$, the planner uses $\theta^{A}=\left(\alpha_{0},\beta_{0},\gamma_{0}\right)$ and $T_{pred}^{A}$. When $d_{target}<d_{th}$, it uses $\theta^{T}=\left(\gamma_{0},\beta_{0},\alpha_{0}\right)$ and $T_{pred}^{T}$, thereby prioritizing velocity in the terminal region while preserving the same clearance weight. The memory coefficient is set to δ=0 in open space and increased to δ=0.15 after an oscillation trigger; it is reset when the UAV leaves the recent-history neighborhood. This rule avoids online parameter search and keeps the DWA scoring function linear at runtime.

### 4.4. Automatic Parameter Tuning Based on Bayesian Optimization

Given the high sensitivity of the dynamic capture performance to the weight coefficients (α,β,γ) in the DWA evaluation function, this paper introduces a Bayesian Optimization (BO) framework. Compared to traditional manual trial-and-error, this mechanism facilitates efficient, global offline searches across a multidimensional parameter space to secure the optimal configuration.

#### 4.4.1. Gaussian Process Surrogate Model Construction

Bayesian optimization models the DWA’s performance in complex pursuit tasks as a black-box objective function f(θ) concerning the parameter vector θ. Given the high computational cost of directly evaluating f(θ) via high-frequency Monte Carlo simulations, a Gaussian Process (GP) is employed as a surrogate model for approximation:

(13) $$f\left(\theta \right)∼GP\left(\mu \left(\theta \right),\kappa \left(\theta ,\theta^{\prime }\right)\right)$$

By learning the spatial correlation of observed sample points, the GP infers the probability distribution in unexplored regions. Its predictive mean μ(θ) represents expected performance, while the uncertainty σ(θ) provides a confidence metric for the search, guiding the algorithm to sample efficiently in potentially optimal regions.

#### 4.4.2. Acquisition Function and Exploration-Exploitation Balance

To resolve the “exploration” versus “exploitation” tradeoff during parameter searching, this study adopts Expected Improvement (EI) as the acquisition function. By quantifying the expected enhancement a potential sampling point might offer over the current optimal observation $f^{*}$, EI determines the next set of parameters to evaluate:

(14) $$\mathrm{EI}\left(\theta \right)=\left(\mu \left(\theta \right)-f^{*}\right)\Phi \left(Z\right)+\sigma \left(\theta \right)\phi \left(Z\right)$$

where $Z=\frac{\mu \left(\theta \right)-f^{*}}{\sigma (\theta )}$, Φ(·) and ϕ(·) denote the cumulative distribution function and probability density function of the standard normal distribution, respectively. By prioritizing parameter combinations with larger EI values, the algorithm effectively escapes local optima and accelerates convergence toward the global solution.

#### 4.4.3. Optimization Objective Definition and Constraint Dimensionality Reduction

The optimization objective is defined as maximizing the distance of the interception location from the safe boundary while minimizing the time required to achieve the capture, thereby ensuring the most efficient cooperative interception. To strictly adhere to the weight normalization constraint α+β+γ=1, a dimensionality reduction mapping method is introduced. By mapping the 3D constrained space onto two independent variable intervals $x_{1},x_{2}\in \left[0,1\right]$, the following relationship is established:

(15) $$\left\{\begin{array}{c} \alpha =x_{1}\\ \beta =x_{2}\cdot \left(1-x_{1}\right)\\ \gamma =1-\alpha -\beta \end{array}\right.$$

This mapping theoretically guarantees the completeness of the parameter space and the efficiency of the search. Experiments demonstrate that the weight configuration, obtained after 500 optimization iterations, enables the UAV to maintain exceptionally high pursuit efficiency even under perceptual noise.

The BO configuration is summarized in Table 3. Failed captures were assigned a large penalty in the objective so that unsafe or non-convergent parameter sets were naturally avoided by the acquisition function. The objective value minimized during BO was defined as

(16) $$J=T_{cap}+0.2L_{path}+5I_{col}+100I_{fail}-0.5d_{min},$$

where $T_{cap}$ is the capture time, $L_{path}$ is the path length, $I_{col}$ and $I_{fail}$ are collision and failure indicators, respectively, and $d_{min}$ is the final distance from the safe boundary.

## 5. Numerical Simulations and Real-World Experiments

This section distinguishes numerical simulations from real-world experiments. The numerical simulations validate repeatability, local-minimum escape, statistical performance, and ablation behavior under controlled randomization. The real-world experiments then examine deployment feasibility on the micro-UAV platform in a suburban environment.

The numerical simulations, Bayesian optimization, and data processing were implemented using MATLAB (R2023b) and Python (v3.10).

This section aims to validate the advantages of the proposed AC2F algorithm in resolving local minima (deadlocks), improving terminal capture precision, and reducing computational complexity through a multi-level evaluation design.

### 5.1. Parameter Tuning Experiment: Bayesian Optimization Results

Section 5.2 (U-shaped trap) and Section 5.3 (dense obstacles) were selected as typical evaluation scenarios to automatically tune the key weights of the DWA using the Bayesian Optimization (BO) framework described in Section 4.4. To strictly satisfy the physical constraint α+β+γ=1 during the optimization process, a dimensionality reduction mapping operator was employed to transform the 3D weight search into a 2-DOF search for independent variables $x_{1},x_{2}\in \left[0.01,0.99\right]$. In each iteration, the optimizer recommended a variable pair $\left(x_{1},x_{2}\right)$, which the simulation system then restored to a specific weight configuration to run a Monte Carlo simulation. This design provided the Gaussian Process (GP) surrogate model with richer topographical information, significantly accelerating convergence. Based on varying convergence rates, Scenario 1 and Scenario 2 underwent 200 and 500 optimization iterations, respectively, to secure their global optimal parameter configurations.

#### 5.1.1. Optimization Results for Scenario 1: Parameter Sensitivity Analysis in a U-Shaped Trap

**Figure 5:**
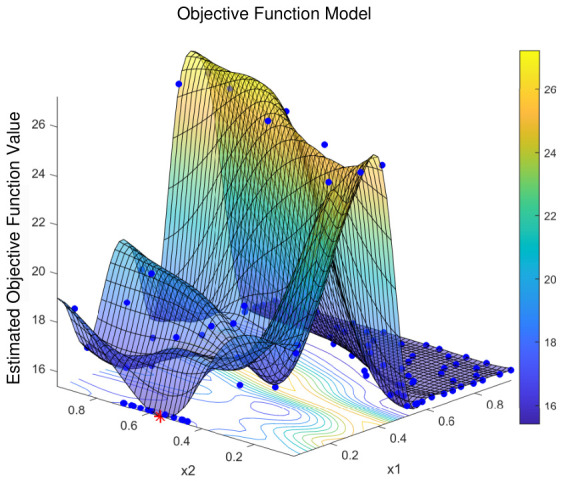
3D Response Surface of the objective function in the U-shaped trap scenario. Blue circles denote evaluated parameter samples, and the red asterisk marks the optimum identified by Bayesian optimization.

In the U-shaped trap scenario, the UAV must strike a delicate balance between “target approach” and “obstacle avoidance.” If the target-oriented weight is too high, the UAV easily falls into a local minimum (deadlock); if the obstacle-avoidance weight is excessive, the trajectory becomes overly conservative, potentially preventing entry into narrow passages. We observed the parameters most sensitive to the deadlock phenomenon. Figure 5 illustrates the 3D response surface based on the reduced variables $x_{1}$ and $x_{2}$. The response surface clearly exhibits a “canyon-like” distribution.

When $x_{1}$ (representing the heading weight) is large (>0.3), the cost function value climbs rapidly above 20, often resulting in capture failure. This indicates that inside the trap, a greedy strategy that forces the UAV to point directly at the target causes it to become stuck at the bottom of the U-shape.

The BO [10,20] rapidly locked the search range into the low-$x_{1}$ region. As summarized in Table 4, after 100 iterations, the algorithm converged to the optimal solution with an Objective = 15.516. The optimal parameter combination at this point was α=0.0502, β=0.6263, and γ=0.3235. This configuration reveals the critical mechanism for escaping a U-shaped trap: by drastically suppressing the heading weight (α≈5%) and significantly elevating the clearance weight (β≈62%), the DWA planner effectively weakens the intention to “rush directly at the target.” Instead, it utilizes the obstacle’s repulsive field to “slide” along the wall edges until it bypasses the U-shaped opening, subsequently relying on the velocity term to pursue the target.

#### 5.1.2. Optimization Results for Scenario 2: Parameter Sensitivity Analysis in Dense Obstacles

**Figure 6:**
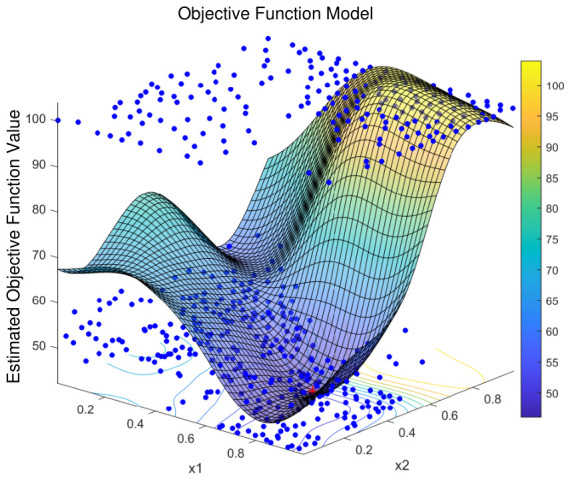
3D Response surface of the objective function in the dense obstacle scenario. Blue circles denote evaluated parameter samples, and the red asterisk marks the optimum identified by Bayesian optimization.

In dense obstacle environments, the topological structure exhibits high randomness and fragmentation. The UAV must not only avoid collisions but also maintain a continuous lock on a high-speed target through fleeting gaps between obstacles. The optimization iteration curve in Figure 6 shows that the search difficulty in this scenario is significantly higher than in Scenario 1. During the first 50 iterations, parameter mismatches frequently led to capture failures (Cost≈100) or excessively long pursuit times. As the GP [11] surrogate model continuously refined its predictions, the algorithm discovered the global optimum (Objective = 42.355) near the 415th generation.

Unlike the U-shaped scenario, the optimal parameters in the dense environment exhibit characteristics of “aggressive tracking”:

In the optimal configuration summarized in Table 5, the heading weight dominates. This demonstrates that in cluttered environments, the UAV must maintain a strong directional lock on the target to closely follow it through obstacle clusters, avoiding the loss of line-of-sight caused by over-evasion.

The obstacle avoidance weight is compressed to an extremely low level (approximately 5%). Data analysis indicates that in dense clutter, an excessively high β induces “hesitant” behavior, causing the UAV to frequently decelerate or make large detours in front of obstacles, thereby missing critical capture opportunities. The low-β strategy encourages the UAV to execute extreme trajectory crossings close to obstacles. Although this slightly increases theoretical risk, it buys crucial pursuit time. To prevent potential collisions caused by significant execution errors in practical engineering applications, a 0.5 m buffer zone is automatically reserved during path calculation.

### 5.2. Qualitative Analysis: Escaping Local Minima

This experiment aims to verify the escape capabilities of the “Oscillation Detection” trigger mechanism (detailed in Section 4.1) and the Fine stage kinodynamic planning strategy within the AC2F framework when facing typical physical deadlocks. A typical “U-shaped trap” scenario was constructed, with the opening facing away from the target point. Initially, the pursuer is located inside the trap, while the target is outside. Under identical initial conditions, we compared the trajectory performance of the traditional Artificial Potential Field (APF) with the proposed AC2F framework.

**APF (Blue Trajectory-Trapped in Deadlock):**

**Figure 7:**
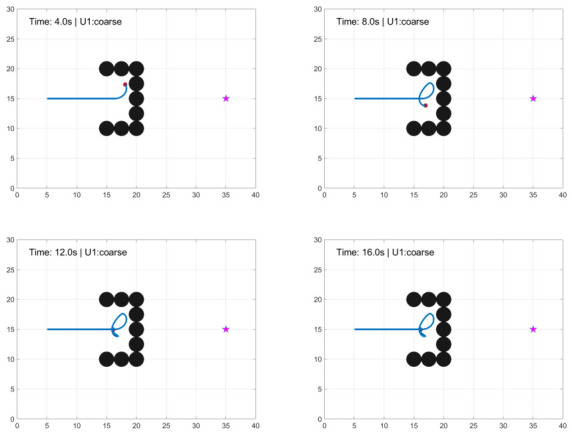
Trajectory of APF trapped in a deadlock. The blue curve shows the pursuer trajectory, the red circle marks its current position, the five-pointed star denotes the target, and the black circles represent obstacles.

As shown in Figure 7, the traditional APF algorithm exhibits inherent limitations when navigating such non-convex obstacles. Guided by the attractive force toward the target, the UAV drives deep into the bottom of the trap. As it approaches the U-shaped base, the outward-pointing attractive force $F_{att}$ and the resultant repulsive force $F_{rep}$ from the walls reach equilibrium along the same collinear axis ($F_{total}\approx 0$). Consequently, the UAV falls into a local minimum, resulting in high-frequency oscillation or stagnation at the bottom, unable to autonomously break the potential field symmetry to navigate out of the trap.

**AC2F (Orange Trajectory-Successful Escape):**

**Figure 8:**
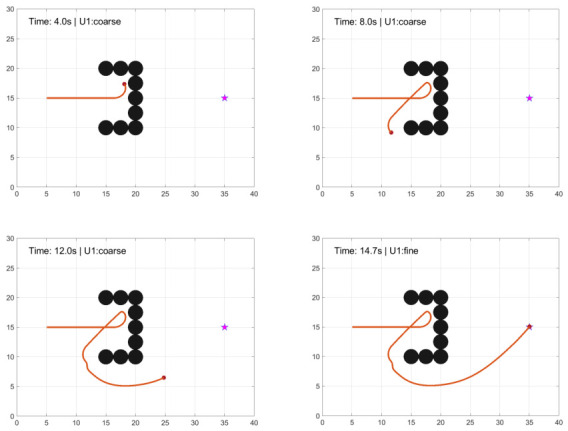
Trajectory of AC2F successfully escaping the U-shaped trap. The orange curve shows the pursuer trajectory, the red circle marks its current position, the five-pointed star denotes the target, and the black circles represent obstacles.

As shown in Figure 8, the AC2F framework successfully achieves escape through the following mechanisms:

- **Mode Switching:** When the UAV falls into a deadlock and experiences in-place oscillation, its positional variance $\sigma^{2}$ drops below the preset sensitivity threshold. The system immediately activates the Oscillation Trigger, switching the control mode from Coarse (geometric potential field guidance) to Fine (kinodynamic local planning).
- **Memory Repulsion Effect:** At this point, the DWA planner in the Fine stage takes over. Crucially, the evaluation function incorporates a Memory Penalty term (see Section 4.3.1). This term imposes a severe penalty on historical positions recently visited by the UAV, essentially compensating for the local minimum within the deadlock zone via an artificially elevated cost.
- **Trajectory Replanning:** As depicted by the orange trajectory, under the combined influence of the memory penalty and collision prediction, the optimal velocity sampling point no longer directs the UAV toward the bottom of the trap. Instead, it points toward the lateral opening of the obstacle. The UAV rapidly executes a sweeping maneuver, flying tangentially along the obstacle edge, ultimately bypassing the U-shaped trap and re-engaging the target.

The experimental results intuitively demonstrate that the proposed “dual-layer state machine” combined with the “historical memory penalty” strategy effectively breaks the mechanical equilibrium that traps traditional potential field methods within regular obstacles. Compared to single-tier planning, this framework endows the system with autonomous decision-making and escape capabilities in complex deadlock environments while maintaining the advantage of low computational overhead.

### 5.3. Quantitative Analysis: Pursuit in Complex Obstacle Environments

To quantitatively evaluate the robustness of the AC2F algorithm in dynamic, unstructured environments, large-scale repeated experiments were conducted within a 75 m × 50 m area containing randomly distributed obstacles. The experiments aimed to verify the algorithm’s capture efficiency, safety, and kinematic smoothness when dealing with initial condition uncertainties.

**Experimental Setup:**

- **Monte Carlo Simulation:** 50 independent simulation rounds were executed.
- **Random Perturbation Injection:** During the initialization phase of each round, random noise of ±1m was applied to the pursuer’s initial position $\left(x_{0},y_{0}\right)$, and a random rotation of ±π/4 was applied to the initial heading $\theta_{0}$. Simultaneously, Gaussian white noise was superimposed on the evader’s decision logic to simulate irrational evasive behavior.
- **Control Group:** A baseline algorithm combining traditional geometric Apollonius circle guidance with APF (Geo + APF) was utilized for comparison.

**Figure 9:**
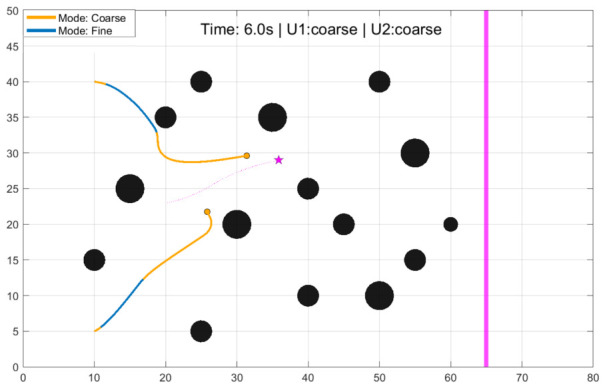
Single detailed simulation trajectory of the AC2F algorithm (6 s). Orange and blue trajectory segments denote the coarse and fine modes, respectively; no tracking-mode segment is present at this time. The magenta dotted line shows the evader trajectory, the five-pointed star marks the current evader position, the magenta vertical line denotes the escape boundary, and black circles of different sizes represent obstacles with different radii.

**Figure 10:**
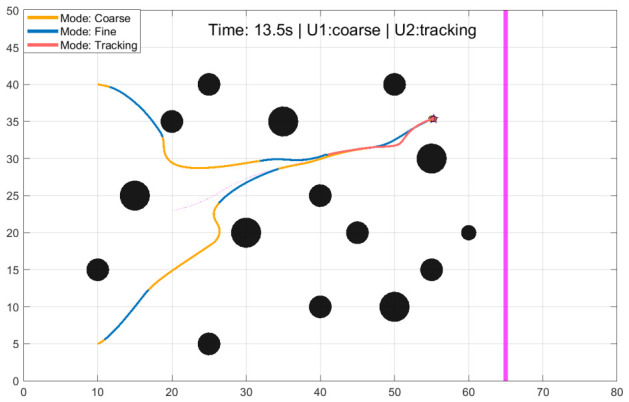
Single detailed simulation trajectory of the AC2F algorithm (13.5 s). Orange, blue, and red trajectory segments denote the coarse, fine, and tracking modes, respectively. The magenta dotted line shows the evader trajectory, the five-pointed star marks the current evader position, the magenta vertical line denotes the escape boundary, and black circles of different sizes represent obstacles with different radii.

The strategic advantages of AC2F can be directly observed through single detailed simulations and multiple superimposed simulations:

As shown in Figure 9 and Figure 10, the AC2F algorithm demonstrates clear state transitions. Orange Trajectory (Coarse Mode): In open areas, the UAV approaches rapidly using APF, incurring low computational overhead. Blue Trajectory (Fine Mode): Upon entering dense obstacle zones (e.g., coordinates x∈[10,38]) or detecting oscillation, the change-detect module activates the fine planning mode. Here, the DWA-based local planner successfully guides the UAV through narrow gaps, preventing the local minimum traps that plague traditional APF in complex force fields. Red Trajectory (Tracking Mode): Executes precise terminal capture. The red tracking segment appears only in Figure 10, because neither UAV had entered the tracking mode at the 6 s instant shown in Figure 9.

**Figure 11:**
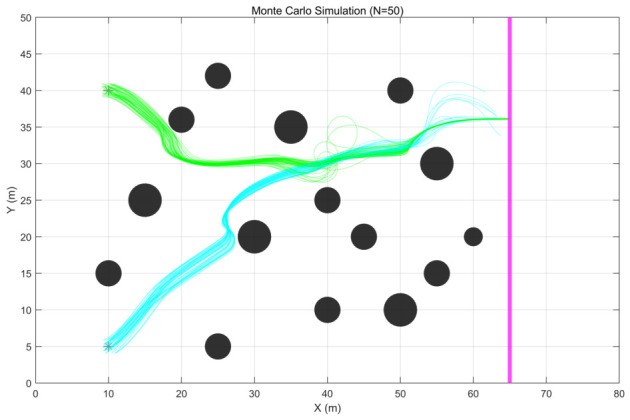
Path superposition of 50 Monte Carlo simulations. Cyan and green curves show the trajectories of the two pursuers, asterisks mark their initial positions, black circles of different sizes represent obstacles with different radii, and the magenta vertical line denotes the escape boundary.

Figure 11 displays the path superposition of 50 Monte Carlo simulations. Despite significant random perturbations in initial positions and evasive strategies, the paths of the two UAVs (cyan and green trajectory bundles) exhibit high consistency. This indicates that the AC2F algorithm possesses robust anti-interference capabilities, consistently generating similar optimal pursuit paths under varying initial conditions rather than diverging into chaotic trajectories.

To objectively evaluate algorithmic performance, four key statistical metrics were defined, and a comparative analysis was conducted based on data recorded from 50 random numerical simulations (summarized in Table 6). For the binary success metric, 95% Wilson confidence intervals were calculated from the number of successful captures. A two-proportion test was also conducted between Geo + APF and AC2F, using 32/50 and 43/50 successful trials, respectively. For continuous metrics, the mean value is reported over successful trials because the archived summary table contains aggregate values rather than all per-run samples.

Quantitative evaluations reveal that the proposed AC2F framework significantly outperforms the Geo + APF baseline across all key metrics. By employing an oscillation detection mechanism to bypass zero-force traps, AC2F increases the capture success rate from 64% to 86%. This corresponds to a 22-percentage-point absolute improvement, a relative success-rate ratio of 1.34, and an odds ratio of 3.46; the two-proportion test indicates statistical significance for the binary capture outcome (p=0.011). Its forward-looking predictive planning eliminates ineffective trial-and-error maneuvers, reducing the average capture time by 0.61s. Furthermore, the integration of DWA’s kinematic window ensures physically continuous and differentiable trajectories, which decreases the average path jerk from $11.05\;{m/s}^{3}$ to $8.69\;{m/s}^{3}$ and effectively reduces motor payload. Finally, AC2F achieves a proactive “forward-pressing” pursuit, successfully intercepting the evader further from the escape boundary (increasing the average safe distance, $d_{min}$, from 7.32 m to 9.07 m) to ensure a significantly higher margin of safety redundancy for the system.

In summary, the AC2F algorithm successfully overcomes the local minimum defects of traditional APF in complex environments through its multi-modal switching strategy. Compared to the baseline algorithm, the proposed scheme not only substantially increases the mission success rate but also achieves an excellent balance between execution efficiency (shorter time) and control quality (smoother paths), validating its superiority in obstacle-dense pursuit tasks.

In addition to Geo + APF, a fixed-weight DWA baseline was included to separate the effect of kinodynamic prediction from the effect of adaptive scheduling. The fixed-weight DWA used the BO-derived approach-mode weights throughout the entire pursuit and did not perform terminal weight swapping or oscillation-triggered memory amplification. As shown later in the ablation study, this baseline improves smoothness relative to APF but still suffers from terminal hesitation and occasional local myopia, indicating that the full gain of AC2F comes from the combined bidirectional switching and adaptive weighting rather than from DWA alone.

### 5.4. Ablation Study: Efficacy of Adaptive Weighting

To verify the necessity of the adaptive weighting mechanism (proposed in Section 4.3.2) in eliminating terminal “tracking error,” an ablation study focusing on the final tracking phase (the last 20 m) was conducted. The experiment simulated a noisy onboard sensor environment to compare the distance convergence performance of a fixed-weight strategy (Blue) against the proposed adaptive-weight strategy (Red).

**Figure 12:**
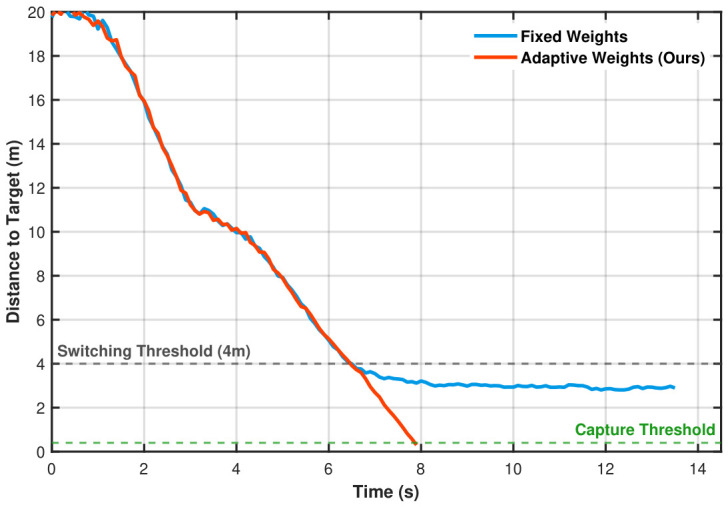
Distance convergence comparison between fixed and adaptive weight strategies.

As shown in Figure 12, the experiment begins 20 m away from the target. During the approach phase at t<6.5s (d > 4 m), the adaptive mechanism has not yet triggered, and the two curves completely overlap. In this phase, both strategies demonstrate good robustness against sensor noise, with the distance decreasing steadily. However, once the distance drops below the 4 m switching threshold (indicated by the gray dashed line), their behaviors diverge significantly:

- **Fixed Weights Strategy (Blue):** Persisting with a long 1.0 s prediction horizon and a lower velocity weight, the UAV exhibits pronounced “conservatism” at extremely close ranges. The slope of the curve gradually flattens after t > 7 s, and the distance error stagnates around 3 m for an extended period. This typical “Trailing Effect” demonstrates that, without parameter adjustments, the UAV struggles to overcome relatively static steady-state errors, failing to meet the 0.4m capture condition even at t=13.5s.
- **Adaptive Weights Strategy (Red):** The algorithm swiftly switches to “Terminal Capture Mode” the moment it detects entry into the capture zone (d < 4 m). By shortening the prediction horizon to 0.2 s and drastically increasing the velocity weight γ (from 0.31 to 0.64), the algorithm sacrifices a degree of path smoothness in exchange for instantaneous explosiveness. This is reflected in the red curve as a sudden increase in the decay rate after 6.5 s, decisively driving the distance toward zero and successfully crossing the capture threshold at t≈8.0s.

The experimental results confirm that during the critical final stage of a high-speed pursuit, incorporating a distance-based dynamic weight-switching mechanism effectively eliminates the terminal “hesitation” inherent in traditional DWA, achieving rapid and precise capture of the escaping target.

A module-wise ablation summary is provided in Table 7. The comparison distinguishes four components: analytical coarse guidance, oscillation-triggered switching, DWA-based kinodynamic prediction, and distance-adaptive terminal weights. Removing any single component changes the dominant failure mode, which supports the interpretation that AC2F is an architecture-level contribution rather than a simple replacement of APF by DWA.

### 5.5. Collaborative Pursuit in Real-World Suburban Scenarios

**Figure 13:**
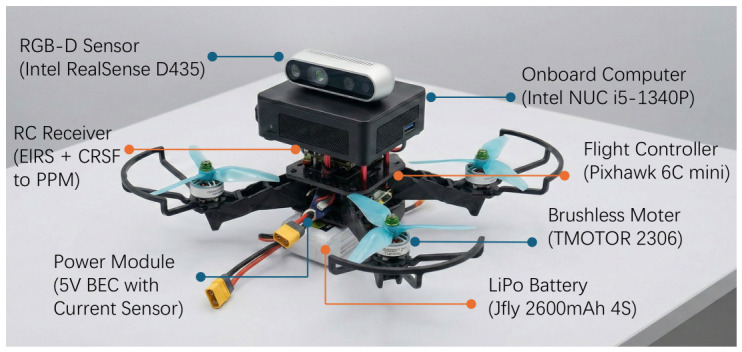
Micro-UAV hardware architecture. The system decouples high-level AC2F decision-making from low-level flight control.

To bridge the gap between simulation and deployment, a custom micro-UAV (Figure 13) was developed for the AC2F framework. It integrates an Intel NUC equipped with an Intel Core i5-1340P processor (Intel Corporation, Santa Clara, CA, USA) for high-level decision-making and DWA planning with an Intel RealSense D435 (Intel Corporation, Santa Clara, CA, USA) for real-time depth sensing. Low-level control is managed by a Pixhawk 6C Mini (Holybro, Hong Kong, China), while the T-MOTOR 2306 propulsion system (T-MOTOR, Nanchang, China) provides the necessary agility for pursuit. This compact integration confirms the algorithm’s effectiveness under the strict computational and physical constraints of micro-platforms.

The sensing and mapping assumptions are as follows. The local obstacle field is obtained from onboard depth sensing and projected onto the horizontal planning plane as inflated obstacle discs using the safety radius in Table 1. The planner does not require a dense pre-built global map during online execution; instead, the coarse layer uses the current pursuer–evader state to update the Apollonius reference point, while the fine layer uses the local obstacle set within the DWA prediction horizon. The evader position is assumed to be available to the cooperative team through onboard perception or communication at the same decision frequency as the planner. These assumptions match the altitude-constrained suburban validation setting, but they also explain why temporary target occlusion and incomplete local perception remain important failure modes.

During deployment, the onboard planner was executed at a 10 Hz decision frequency, matching the DWA control period in Table 1. Runtime logs were used to verify that the fine-stage planner remained within the real-time budget on the Intel NUC without requiring a GPU/NPU accelerator. Table 8 summarizes the onboard computational characteristics of the AC2F implementation under this hardware configuration. Because planning latency is platform-dependent, the reported values are interpreted together with the Intel NUC i5-1340P specification described above.

The quantitative pursuit results obtained in the suburban scenarios are summarized in Table 9.

**Figure 14:**
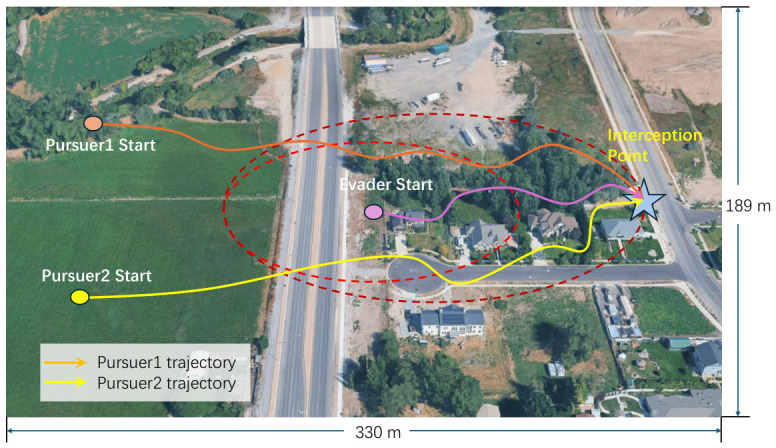
Suburban pursuit case study (330 m × 189 m). Red dashed lines denote Apollonius capture sets, showing the transition from global guidance to fine kinodynamic avoidance.

To further validate the practical engineering feasibility and robustness of the proposed AC2F framework in unstructured environments, a comprehensive real-world case study was conducted in a suburban area measuring 330m×189m. As illustrated in Figure 14, the task involves two pursuer UAVs coordinating to intercept a dynamic evader amidst complex physical obstacles, including residential buildings and dense vegetation. During the coarse guidance phase, the pursuers leverage the analytical Apollonius circle to instantaneously calculate the optimal interception point $P_{global}$ and establish a collaborative capture zone (denoted by the red dashed areas), thereby ensuring global pursuit efficiency with minimal computational overhead. As Pursuer 2 navigates through the cluttered residential cluster, the FSM-based switching mechanism seamlessly transitions the control to the fine stage. By employing the BO-optimized DWA parameters, the UAV successfully executes smooth, kinodynamically feasible maneuvers through narrow gaps, effectively bypassing potential local minima that typically entrap traditional reactive methods. The convergence of trajectories at the predicted interception star demonstrates that the AC2F strategy rigorously balances macroscopic mission efficiency with microscopic flight safety under stringent micro-platform constraints.

These comparative results show that AC2F achieves an 86.4% success rate, significantly outperforming APF (64.2%) and fixed-weight DWA (71.8%). This superiority stems from the bidirectional switching and BO-optimized parameters, which allow the UAV to escape U-shaped suburban traps that typically entrap traditional reactive controllers. The notable reduction in capture time and path length further evidences enhanced mission efficiency. Coupled with its low computational footprint on the Intel NUC, AC2F offers a robust, “engineering-friendly” solution for public domain security and civil surveillance.

## 6. Discussion

### 6.1. Predictive Kinodynamic Planning vs. Instantaneous Reactive Control

The performance gain of AC2F over Geo + APF can be interpreted primarily through the difference between predictive kinodynamic planning and instantaneous reactive control.

APF relies on instantaneous spatial forces and does not explicitly account for the UAV’s velocity or inertia. As a result, obstacle responses may occur only at short range, leading to abrupt deceleration or collision risk. In contrast, the DWA module evaluates feasible future states over a finite horizon, allowing the controller to select trajectories that are both dynamically executable and consistent with the current motion state.

### 6.2. Engineering Feasibility and Practical Significance

Although Deep Reinforcement Learning (DRL) [4,21] has shown strong performance in end-to-end navigation, AC2F provides a more transparent alternative for resource-constrained micro-UAV deployment.

When planning fails, the evaluation weights, sampled trajectories, and FSM transitions can be inspected directly. This traceability supports debugging and safety verification, whereas the internal decision process of learned policies is typically less transparent.

DRL algorithms often require GPUs or NPUs for real-time neural-network inference [5,6]. AC2F instead follows an “offline optimization, online execution” paradigm: Bayesian optimization is performed before deployment, whereas onboard computation is limited to analytical geometric guidance, finite-state switching, and linear DWA trajectory scoring [22].

The practical significance of this architecture lies in its selective use of computation. Low-cost geometric guidance handles most pursuit decisions, while the DWA planner is activated only near terminal capture or when oscillation indicates local trapping. This division is relevant to public-domain pursuit tasks, where micro-UAVs must balance obstacle response, onboard resource limits, and interpretable control behavior.

### 6.3. Parameter Sensitivity and Failure Cases

The distance threshold and oscillation variance threshold determine when the planner enters the fine stage. In the current implementation, $d_{th}=4.0$ m is larger than the capture radius so that the DWA module can reduce terminal trailing error before interception. Smaller values may delay the switch, whereas larger values may invoke DWA too often and weaken the computational advantage of the coarse stage. Similarly, $\epsilon_{osc}=0.08$ m^2^ is set below the variance observed during normal curved tracking but above the near-zero variance observed during trap-induced stagnation.

The main failure cases are temporary target loss under depth-sensing occlusion, conservative detours when no dynamically feasible velocity sample remains in dense obstacles, and missed terminal synchronization when one pursuer reaches the tracking state much earlier than the other. These cases indicate that future versions should integrate stronger local mapping and multi-agent timing coordination.

### 6.4. Limitations and Future Work

Several limitations remain. The current kinematic derivations and experiments use a 2D planar model, which is suitable for altitude-stratified operations but does not fully represent vertical maneuvering. Extending the framework to 3D would require vertical velocity or climb-rate constraints, volumetric collision checking, and a three-dimensional form of the Apollonius reachable set. The current evader model also mainly moves toward the nearest safe boundary. Future work will therefore examine more complex non-cooperative behaviors together with fully 3D local planning.

## 7. Conclusions

This paper proposed a lightweight Adaptive Coarse-to-Fine (AC2F) hybrid planning strategy for multi-UAV cooperative pursuit in obstacle-dense environments. The framework integrates Apollonius-circle-based global guidance with DWA-based local kinodynamic planning through bidirectional state switching.

The primary contributions and conclusions are summarized as follows:

- **Resolving the Conflict Between Efficiency and Safety:** Through dynamic finite-state machine scheduling and adaptive weight adjustments, the UAV invokes high-fidelity, computationally intensive obstacle avoidance strictly when necessary. This dynamically balances the macroscopic efficiency of global interception with the microscopic safety of local flight.
- **Overcoming Deadlock Vulnerabilities:** Experimental results validate that the integration of DWA’s forward-looking prediction, coupled with a historical memory penalty, effectively resolves the local minimum flaws inherent in traditional potential field methods. This mechanism successfully navigates challenging topologies, such as U-shaped traps, increasing the overall capture success rate from 64% to 86% compared to baseline methods.
- **Engineering-Friendly Deployment:** Offline Bayesian Optimization automates the calibration of critical evaluation parameters and reduces manual tuning. Combined with the low online computational footprint of the coarse-to-fine architecture, AC2F is suitable for resource-constrained micro-UAV platforms.

Future work will focus on extending this algorithm to fully three-dimensional spaces and integrating onboard perception-based local mapping techniques to further enhance the system’s autonomous capabilities in entirely unknown environments. 

## Figures and Tables

**Table 1:** Implementation parameters of the AC2F planner.

Parameter	Symbol	Value	Role
Fine-stage distance trigger	$d_{th}$	4.0 m	Enters terminal kinodynamic planning near the evader.
Oscillation window	$t_{w}$	2.0 s	Sliding window for local-minimum detection.
Oscillation variance threshold	$\epsilon_{osc}$	0.08 m^2^	Activates DWA when recent motion is confined.
Fine-stage maximum duration	$T_{fine}$	3.0 s	Forces return to coarse guidance after local escape.
DWA control period	Δt	0.1 s	Online replanning interval.
Prediction horizon, approach mode	$T_{pred}^{A}$	1.0 s	Longer look-ahead for stable tracking.
Prediction horizon, terminal mode	$T_{pred}^{T}$	0.2 s	Shorter look-ahead for close-range capture.
Safety saturation distance	$D_{sat}$	2.0 m	Upper bound for clearance reward.
Collision rejection radius	$R_{collision}$	0.5 m	Hard safety radius for infeasible trajectories.
History length for memory penalty	$N_{h}$	30 samples	Recent positions used to penalize looping trajectories.
BO initialization/evaluation budget	–	20/200–500	Random initial samples and scenario-dependent BO iterations.

**Table 2:** Finite-state switching logic of AC2F.

State	Condition and Action	Next State
Coarse	Compute $P_{global}$ from Apollonius guidance and apply lightweight APF avoidance. If $d_{target}<d_{th}$ or $\sigma_{p}^{2}<\epsilon_{osc}$ over $t_{w}$, activate DWA.	Fine
Fine	Sample admissible (v,ω), reject collisions, evaluate G(v,ω) with the memory penalty, and execute the best command. If capture is satisfied but teammates are not ready, hold relative position.	Tracking
Fine	If $T_{fine}$ expires before capture, return to geometric guidance to restore global pursuit intent.	Coarse
Tracking	Maintain relative position until cooperative encirclement is completed or the evader leaves the capture zone.	Coarse/Fine

**Table 3:** Bayesian optimization configuration.

Item	Configuration
Search variables	$x_{1},x_{2}\in \left[0.01,0.99\right]$, mapped to normalized (α,β,γ).
Surrogate model	Gaussian Process with a squared-exponential covariance kernel.
Acquisition function	Expected Improvement (EI).
Initial samples	20 Latin-hypercube random samples.
Evaluation budget	200 iterations for the U-shaped scenario and 500 iterations for dense obstacles.
Failure handling	Collision or no capture within the time limit receives $I_{fail}=1$.
Output	Scenario-specific baseline weights used by the onboard DWA planner.

**Table 4:** Performance Comparison Before and After Optimization in Scenario 1.

Parameter Configuration	α (Heading)	β (Clearance)	γ (Velocity)	Cost	Result Analysis
Initial Random Set	0.2234	0.7091	0.0675	18.56	pursuit is slow.
Typical Deadlock Set	0.3440	0.4032	0.2528	Fail	trapped at the bottom
Bayesian Optimal Set	0.0502	0.6263	0.3235	15.52	Optimal solution

**Table 5:** Performance Comparison Before and After Optimization in Scenario 2.

Parameter Configuration	α (Heading)	β (Clearance)	γ (Velocity)	Cost	Result Analysis
Safety-Biased Set	0.4829	0.5019	0.0152	Fail	Target lost
Balanced Weight Set	0.2123	0.0551	0.7326	59.20	poor directional focus
Bayesian Optimal Set	0.6392	0.0490	0.3118	42.36	Optimal solution

**Table 6:** Key statistical metrics under different planning strategies in 50 numerical simulations.

Evaluation Metric	Geo + APF (Control)	AC2F (Proposed)	Improvement
Success Rate (95% CI)	64% [50.1, 75.9]	86% [73.8, 93.0]	+22 points; p=0.011
Average Capture Time	13.65 s	13.04 s	4.47%
Path Smoothness (Avg. Jerk)	$11.05\;{m/s}^{3}$	$8.69\;{m/s}^{3}$	21.36%
Avg. Distance to Safe Boundary ($d_{min}$)	7.32 m	9.07 m	23.9%

**Table 7:** Module-wise ablation of AC2F components.

Variant	Coarse	Switch	Adapt.	Observed Effect
Geo + APF	Yes	No	No	Low computation, but frequent deadlock in U-shaped and concave obstacles.
Fixed DWA only	No	No	No	Smooth local motion, but weak global pursuit intent and longer detours.
AC2F without memory	Yes	Yes	Yes	Can enter fine mode, but may revisit historical positions in traps.
AC2F without terminal adaptation	Yes	Yes	No	Escapes obstacles, but shows close-range trailing error.
Full AC2F	Yes	Yes	Yes	Balances global interception, local escape, and terminal capture.

**Table 8:** Onboard computational-cost summary for the AC2F implementation.

Metric	Coarse Stage	Fine Stage	Interpretation
Decision frequency	10 Hz	10 Hz	Same controller interface for both stages.
Planning-latency budget	100 ms	100 ms	Upper bound imposed by the control period.
Measured planning latency	<1 ms	<20 ms	DWA remains below the real-time budget on the Intel NUC i5-1340P.
Accelerator requirement	No	No	CPU-only execution; no GPU/NPU inference is required.
Dominant computation	Apollonius geometry	Velocity sampling and trajectory scoring	Heavy BO search is performed offline.

**Table 9:** Quantitative comparison of pursuit performance in suburban scenarios.

Method	Success Rate (%)	Avg. Time (s)	Avg. Path (m)
Traditional APF	64.2	142.5	512.3
Fixed-weight DWA	71.8	128.4	485.6
AC2F (Ours)	86.4	105.2	432.8

## Data Availability

The raw data supporting the conclusions of this article will be made available by the authors on request.

## Abbreviations

The following abbreviations are used in this manuscript:

UAV	Unmanned Aerial Vehicle
AC2F	Adaptive Coarse-to-Fine
DWA	Dynamic Window Approach
BO	Bayesian Optimization
GP	Gaussian Process
APF	Artificial Potential Field
FSM	Finite State Machine
DRL	Deep Reinforcement Learning
LOS	Line-of-Sight
MPC	Model Predictive Control
AGV	Automated Guided Vehicle
